# IL-11 Attenuates Liver Ischemia/Reperfusion Injury (IRI) through STAT3 Signaling Pathway in Mice

**DOI:** 10.1371/journal.pone.0126296

**Published:** 2015-05-06

**Authors:** Miao Zhu, Bo Lu, Qinhong Cao, Zhenfeng Wu, Zhe Xu, Weisu Li, Xuequan Yao, Fukun Liu

**Affiliations:** 1 Department of Surgical Oncology, Affiliated Hospital of Nanjing University of TCM, 155 Hanzhong Road, Nanjing, Jiangsu Province, P. R China; 2 Department of General Surgery, Yixing People’s Hospital, 75 Tongzhenguan Road, Yixing, Jiangsu Province, P. R China; Virginia Commonwealth University, UNITED STATES

## Abstract

**Background:**

The protective role of IL-11, an IL-6 family cytokine, has been implicated in ischemia/reperfusion injury (IRI) in the heart and kidney, but its role has not been elucidated in liver IRI. This study was designed to evaluate the effects of IL-11 and its mechanism of action on liver IRI in a mouse model.

**Methods:**

A partial (70%) warm liver IRI was induced by interrupting the artery/portal vein blood supply to the left/middle liver lobes. IL-11 mRNA expression of ischemic liver after reperfusion was analyzed. Signal transducer and activator of transcription 3 (STAT3) was analyzed following IL-11 treatment in vivo and in vitro. Next, IL-11 was injected intraperitoneally (ip) 1 hour before ischemia. Liver injury was assessed based on serum alanine aminotransferase levels and histopathology. Apoptosis and inflammation were also determined in the ischemic liver. To analyze the role of STAT3 in IL-11 treatment, STAT3 siRNA or non-specific (NS) siRNA was used in vitro and in vivo.

**Results:**

IL-11 mRNA expression was significantly increased after reperfusion in the ischemic liver. STAT3, as a target of IL-11, was activated in hepatocytes after IL-11 treatment in vivo and in vitro. Next, effects of IL-11/STAT3 signaling pathway were assessed in liver IRI, which showed IL-11 treatment significantly attenuated liver IRI, as evidenced by reduced hepatocellular function and hepatocellular necrosis/apoptosis. In addition, IL-11 treatment significantly inhibited the gene expressions of pro-inflammatory cytokines (TNF-α and IL-10) and chemokines (IP-10 and MCP-1). To determine the role of STAT3 in the hepatoprotective effects of IL-11, STAT3 siRNA or NS siRNA was used prior to IL-11 treatment. The results showed STAT3 knockdown abrogated the protective effects of IL-11 in vitro and in vivo.

**Conclusions:**

This work provides first-time evidence for the protective effect of IL-11 treatment on hepatocyte in liver IRI, through the activation of the STAT3 pathway.

## Introduction

Ischemia-reperfusion injury (IRI) is a key contributing factor in liver dysfunction and failure after hepatic trauma, resection, liver transplantation, and circulatory shock [1–4]. An effective method for preventing or minimizing liver IRI is urgently needed in liver surgery. The factors/pathways have been involved in the hepatic IRI process include anaerobic metabolism, mitochondria damage, oxidative stress, endoplasmic reticulum stress, intracellular calcium overload, Kupffer cell (KC) activation, neutrophil infiltrations, and production of cytokines and chemokines [1–3]. The adverse factors mentioned above finally lead to cell death/apoptosis, which indicates that cell death/apoptosis is a significant and perhaps principal contributor to liver IRI [5–7]. Thus, understanding the sequence of events central to the cell death/apoptosis mechanism may potentially lead to treatments for liver IRI.

IL-11 is a hematopoietic IL-6 family cytokine with multifunctional effects. Indeed, IL-11 has thrombopoietic activity, and recombinant human IL-11 has been used for thrombocytopenia in clinical settings [8]. Different from other IL-6 family cytokines, IL-11 holds anti-inflammatory function against chronic inflammatory diseases, lipopolysaccharide-induced sepsis, etc [9–11]. Kimura’s group reported that IL-11 played a cardioprotective role, and conferred resistance to heart IRI in a mouse model by enabling significant anti-necrotic/apoptotic effects [12]. In addition, IL-11 pretreatment also reduces IR-induced cell death/apoptosis by up-regulating Bcl-2 [13]. More importantly, IL-11 shares some similar effects to other IL-6 family members. It has been reported that IL-11 binding with gp130 receptor induces activation of STAT3, which is involved in many physiological and pathological processes [14]. Inhibitors of STAT3 phosphorylation or dominant-negative STAT3 mutants facilitate the expression of pro-apoptosis factors, suggesting that STAT3 plays a critical role in regulating cell proliferation and anti-apoptosis [15]. Furthermore, STAT3 knockout mice exhibit complete embryonic lethality [16]. Conditional ablation of STAT3 in myocardial cells leads to higher susceptibility to drug-induced heart failure [17]. To the best of our knowledge, there has been no report on IL-11 preconditioning before liver IRI. In the present study, we tested the hypothesis that exogenous IL-11 attenuates liver IRI by STAT3-mediated anti-necrotic/apoptotic effects.

## Materials and Methods

### Animals

Male C57BL/6 mice were purchased from the Laboratory Animal Resources Center of Nanjing Medical University (NMU). The animals were fed a laboratory diet with water and food and kept under constant environmental conditions with 12h light–dark cycles. Procedures were carried out in accordance with the Guidelines for Laboratory Animal Care. The animal protocol had been approved by the Institutional Animal Care & Use Committee (IACUC) of Nanjing Medical University (Protocol Number NMU08-092).

### Surgical procedure and IL-11 treatment

The present study used a well-established mouse model of partial (70%) warm hepatic IRI [18]. Anesthesia was induced by 10% chloral hydrate (0.3g/kg, intraperitoneally). Mice were injected with heparin (100U/kg), and an atraumatic clip was used to interrupt the artery/portal vein blood supply to the left/middle liver lobes. After 90 minutes of ischemia, the clip was removed, and the mice were sacrificed (cervical dislocation) at required times after reperfusion. Some mice received a single injection of recombinant human IL-IL-11 (500μg/kg, ip) (PeproTech, Rocky Hill, NJ) or medium (PBS) 1 hour prior to ischemia. PBS injection was used as a control. Sham-operated controls underwent the same procedure but without vascular occlusion. To access effects of STAT3 on IL-11 treatment, STAT3 siRNA or NS siRNA (2mg/kg) was given intravenously 4 hours prior to ischemia [19]. Reports have previously documented the efficacy of this siRNA approach in the liver, with>40% of intravenously infused siRNA accumulating in the ischemic mouse livers [20].

### Serum biochemical examination

Blood samples collected 6h after reperfusion was centrifuged to obtain serum. The serum level of alanine aminotransferase (sALT) or supernatant level of lactate dehydrogenase (LDH) was measured to assess the extent of hepatocyte damage using an automated chemical analyzer (Olympus Automated Chemistry Analyzer AU5400, Tokyo, Japan).

### Histopathologic study

Liver specimens were fixed with 10% neutral formaldehyde and then embedded in paraffin. The specimens were sectioned at 4μm and stained with hematoxylin and eosin. The sections were used in histopathologic analysis by light microscopy. Sections were scored from 0 to 4 for sinusoidal congestion, vacuolization of hepatocyte cytoplasm, and parenchymal, as described by Suzuki et al [21].

### Caspase-3 activity assay

Caspase-3 activity was assayed in liver tissues 6h after reperfusion. Frozen samples of ischemic tissues were homogenized with a Polytron homogenizer and centrifuged at 16,000g for 20 minutes. Activity was measured with an assay kit (Calbiochem) according to the manufacturer’s instructions.

### Terminal deoxynucleotidyl transferase dUTP nick end labeling (TUNEL) staining

Paraffin sections (4μm in thickness) were deparaffinized in toluene and then dehydrated in a graded series of ethanol solutions. Sections were stained by TUNEL using a commercially available kit (in situ cell death detection kit, Roche-Boehringer Mannheim, Germany).

### Western blot analysis

Proteins were extracted from liver tissues subjected to ischemia or from cell lysates, and their concentrations were determined by the Bradford assay (Bio-Rad, CA). About 30 μg protein was resolved by sodium dodecyl sulfate polyacrylamide gel electrophoresis and transferred to nitrocellulose membranes (Sunshine Biotechnology, China). These membranes were blocked in skim milk powder (5% wt/vol) with phosphate buffered saline containing 0.1% Tween 20 (PBS-T) at 4°C overnight. Membranes were then incubated with primary antibodies for cleaved Caspase-3, P-STAT3, Bcl-2, Bax, β-actin (Cell Signaling Technology, Danvers, MA), and STAT3 (Santa Cruz Biotechnology, Santa Cruz, CA). Following three washes with PBS-T, the membranes were incubated for 1h at room temperature with peroxidase-conjugated secondary antibody (Cell Signaling Technology, Danvers, MA). The final results were obtained by exposure to autoradiographic film (Kodak XAR film), and then visualized via a chemiluminescent detection system (ECL Substrate Western blot detection system, Pierce, IL).

### Quantitative real-time PCR

Quantitative real-time PCR was performed using the DNA Engine with a Chromo 4 Detector (MJ Research, Waltham, MA). In a final reaction volume of 25μl, the following were added: 1×SuperMix (Platinum SYBR Green qPCR Kit; Invitrogen), cDNA, and 2.5μM of each primer. The amplification conditions were as follows: 50°C (2 min), 95°C (5 min), followed by 50 cycles at 95°C (15 sec) and 60°C (30 sec). The expression of the target genes (IL11, TNF-α, IL-6, IP-10 and MCP-1) (Invitrogen, Shanghai, China) was calculated based on the ratio of the gene of interest to the housekeeping gene HPRT. Primer sets (sense sequence and antisense sequence, respectively) for the following genes were: HPRT forward, 5’- TCA ACG GGG GAC ATA AAA GT-3’, reverse, 5’- TGC ATT GTT TTA CCA GTG TCA A’; IL-11 forward: 5’- CTG CCC ACC TTG GCC ATG AG-3’; IL-11 reverse: 5’- CCA GGC GAG ACA TCA AGA AAG A-3’; TNF-α forward, 5’- GCC TCT TCT CAT TCC TGC TTG T-3’, reverse, 5’- TTG AGA TCC ATG CCG TTG-3’; IL-6 forward, 5’- GCT ACC AAA CTG GAT ATA ATC AGG A-3’, reverse, 5’- CCA GGT AGC TAT GGT ACT CCA GAA-3’; IP-10 forward. 5’-GCT GCC GTC ATT TTC TGC-3’, reverse, 5’-TCT CAC TGG CCC GTC ATC-3’; MCP-1 forward, GGT GAT AAC CGC CCT AGC-3’, reverse, 5’-TGT GTC GGC TGG ATA GGC-3’.

### Cell culture and treatment

Mouse hepatocytes were isolated using a two-step in situ collagenase perfusion procedure [18]. Livers from the C57BL/6 mice were perfused in situ through the portal vein with ethylene glycol tetraacetic acid (EGTA) buffer (0.5mM EGTA, 137mM NaCl, 4.7mM KCl, 1.2mM KH2PO4, 0.65mM MgSO4, and 10.07mM HEPES at pH 7.4) at a flow rate of 5ml/min for 10 min, followed by collagenase buffer (67mM NaCl, 6.7mM KCl, 4.76mM CaCl2, 0.035% collagenase type II, and 10.07mM HEPES at pH 7.6) at a flow rate of 5ml/min for 15 min. After centrifugation, the hepatocytes were collected and seeded in DMEM containing 10% FBS, 100units/ml penicillin, and 100μg/ml streptomycin. Cells were preincubated with IL-11 (1μg/ml for 1h), then H_2_O_2_ (200μm for 24h) to induce cell death.

### Knockdown of STAT3 expression using STAT3 siRNA transfection

Hepatocytes were grown and transiently transfected with STAT3 siRNA or NS siRNA using Transfection Reagent LipofectamineTM RNAiMAX (Invitrogen, CA, USA) according to the manufacturer’s instructions. In brief, cells were seeded at 1 x 10^6^ per well in 1.5ml of OPTI-medium (Invitrogen, CA, USA) in a 6-well plate. After 20h, the cells were transfected with 20nmol/ml STAT3 siRNA or NS siRNA. About 6h after transfection, the medium was changed to a regular medium, and the cells were treated as described above after 24h.

### Statistical analysis

The data are presented as the mean ± SEM from at least three independent experiments. One-way analysis of variance test [—] was used in comparisons of three groups. Student’s t-test [∏] was used for comparison of two groups. All P values were two-sided, and P<0.05 was considered to be statistically significant.

## Results

### IL-11 is elevated in IR-stressed liver

**Fig 1:**
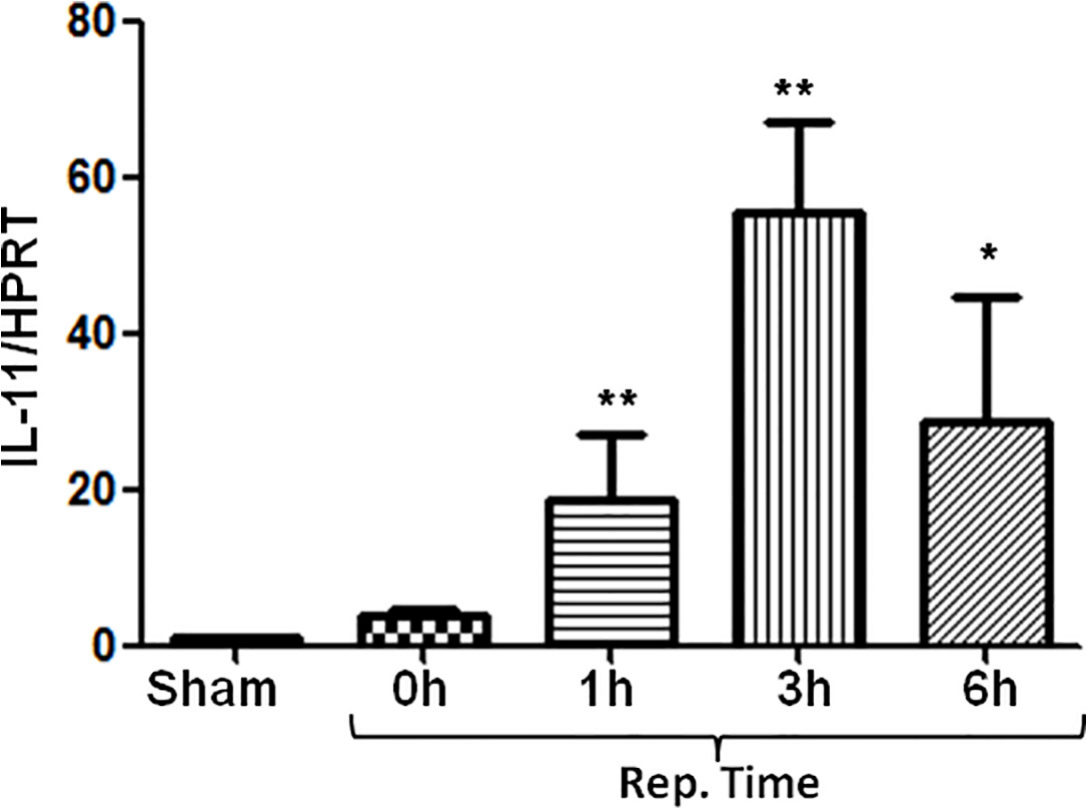
IL-11 expression was increased after IR. Mice were subjected to 90min of partial liver ischemia, followed by 0h, 1h, 3h and 6h reperfusion. Kinetics of IL-11 gene expression was analyzed in ischemic liver by RT-PCR. Expression of IL-11 was normalized with that of HPRT. Data are expressed as mean±SD (n = 6/group). *P<0.05, **P<0.001 vs sham group.

To determine the effects of IL-11 on liver IRI, we first analyzed the gene expression of IL-11 in ischemic livers after various reperfusion time points. As shown in Fig 1, the expression of IL-11 was increased at 0h post reperfusion and reached its peak 3h post reperfusion. The data indicate that IL-11 was present in the liver 3h after reperfusion.

### IL-11 activates STAT3 in liver and hepatocytes

**Fig 2:**
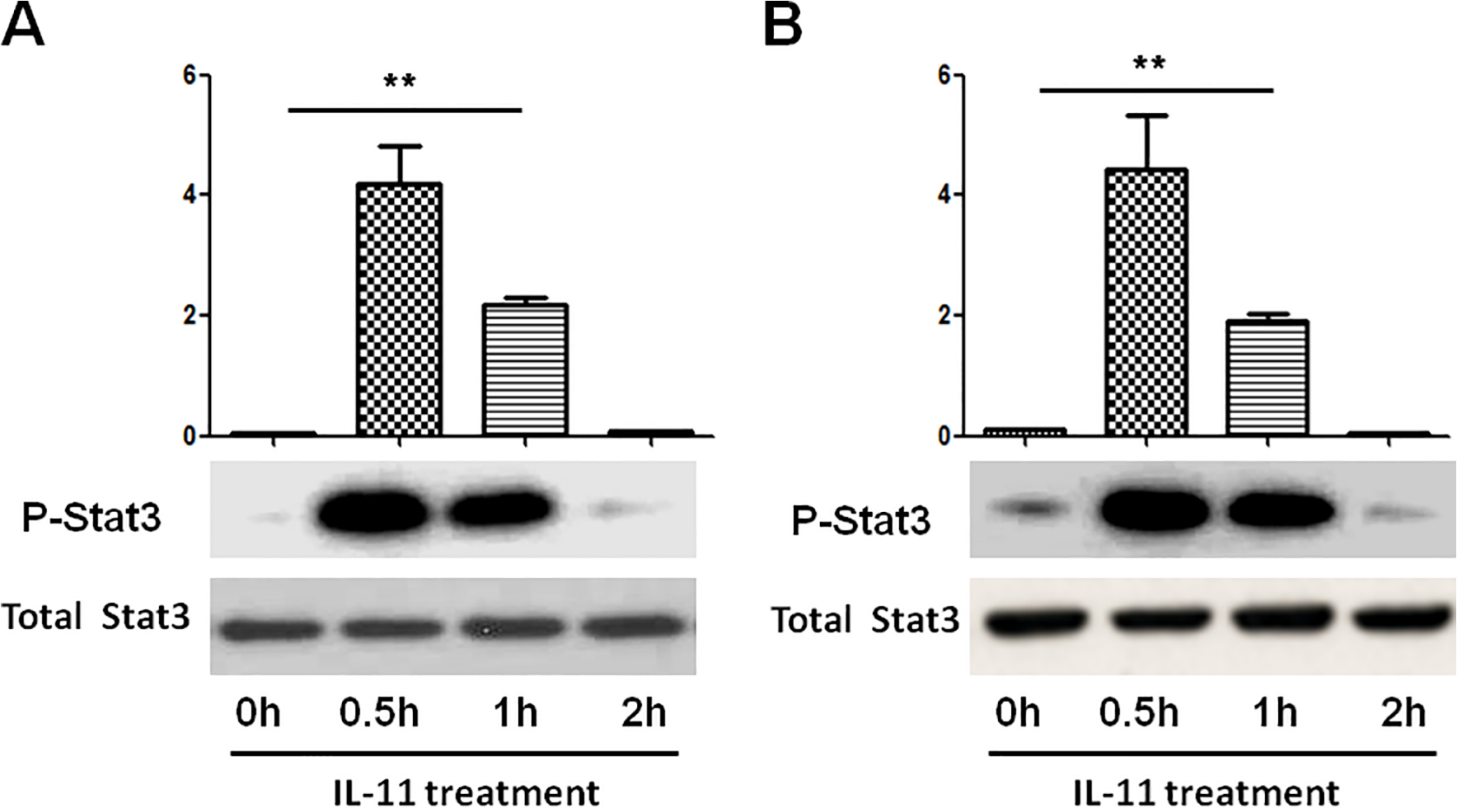
IL-11 activated STAT3 in hepatocytes in vivo and in vitro. (A) IL-11 was administered in mice for the indicated time. The lysates from liver were immunoblotted with anti-p-STAT3 and anti-STAT3 antibody. Representative data are shown (lower). Quantative analyses of p-STAT3 are shown (upper). Data are exprresed as mean±SD (n = 6/group). **P<0.001. (B) Hepatocytes were treated by IL-11 for the indicated time. The lysates from hepatocytes were immunoblotted with anti-p-STAT3 and anti-STAT3 antibody. Representative data are shown (lower). Quantative analyses of p-STAT3 are shown (upper). Data are expressed as mean±SD (n = 6/group), **P<0.001.

IL-11, an IL-6 family cytokine, was supposed to activate STAT3. Here we determined whether administration of IL-11 stimulates STAT3 in liver by western blotting with antibody (Fig 2A). Phosphorylation of STAT3 was rapidly induced and recovered nearly to baseline after 2 hours. To further ascertain that phosphorylation of STAT3 occurred in hepatocytes, we analyzed the effects of IL-11 on STAT3 activity of hepatocytes in vitro. Fig 2B shows phosphorylation of STAT3 was also significantly elevated after IL-11 treatment, indicating that IL-11 administration may activate STAT3 within parenchymal cells in the liver.

### IL-11 attenuates liver IRI

**Fig 3:**
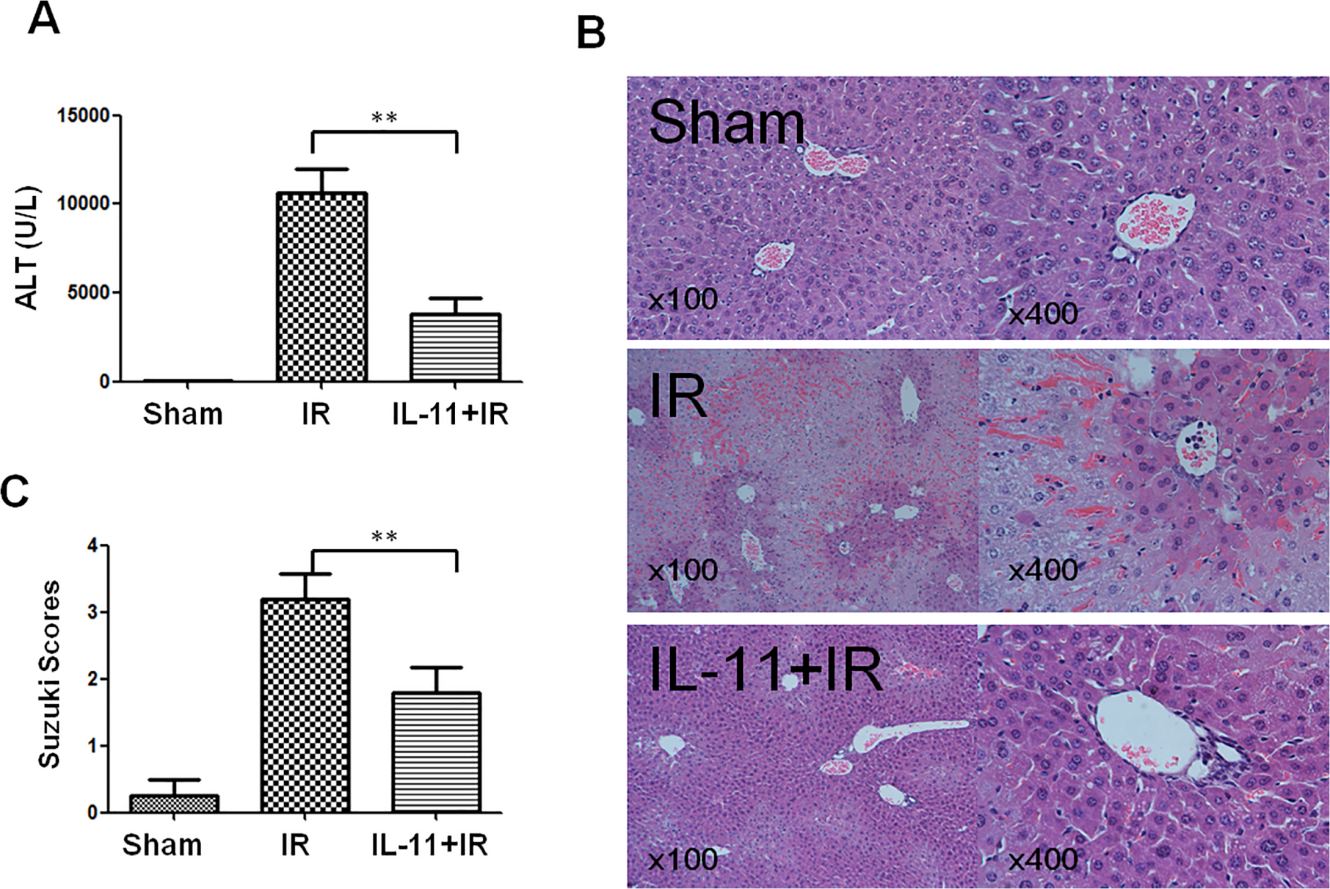
IL-11 treatment attenuated liver injury induced by IR. Mice were administered with recombinant human IL-IL-11 (500μg/kg, ip) or medium (PBS) 1 hour prior to ischemia, followed by 6h reperfusion. (A) sALT. (B) Histopathalogic analysis of livers harvested 6 hours after reperfusion. Sham group: Normal hepatic architecture; IR group: severe hepatic lobule distortion, sinusoidal congestion, apparent edema, vacuolization and massive necrosis; IL-11+IR group: mild vacuolization, punctate necrosis and edeman. (C) The severity of liver IRI by Suzuki’s histological grading. Data are expressed as mean±SD (n = 6/group), **P<0.001.

Next, we analyzed effects of IL-11 administration in liver IRI. Mouse livers were subjected to 90 min of warm ischemia 6h after reperfusion. sALT levels in each group were analyzed (Fig 3A). sALT levels were markedly increased in the IR group compared with that of the sham group (33.33±5.49 and 10610.00±1393.00, respectively; *P*<0.01). By contrast, when mice were pretreated with IL-11, sALT levels (3832.00±834.90, *P*<0.01) were significantly decreased compared with those in the IR control. Liver serum enzyme data were in line with liver pathological analysis (Fig 3B and 3C). The histological parameters in the sham (0.25±0.25), IR (3.20±0.37), and IL-11 administration (1.8±0.37) groups were observed according to Suzuki et al [26]. These data indicate that IL-11 treatment significantly attenuates IR-induced liver injury.

### IL-11 exhibits anti-apoptotic functions and reduces apoptosis

**Fig 4:**
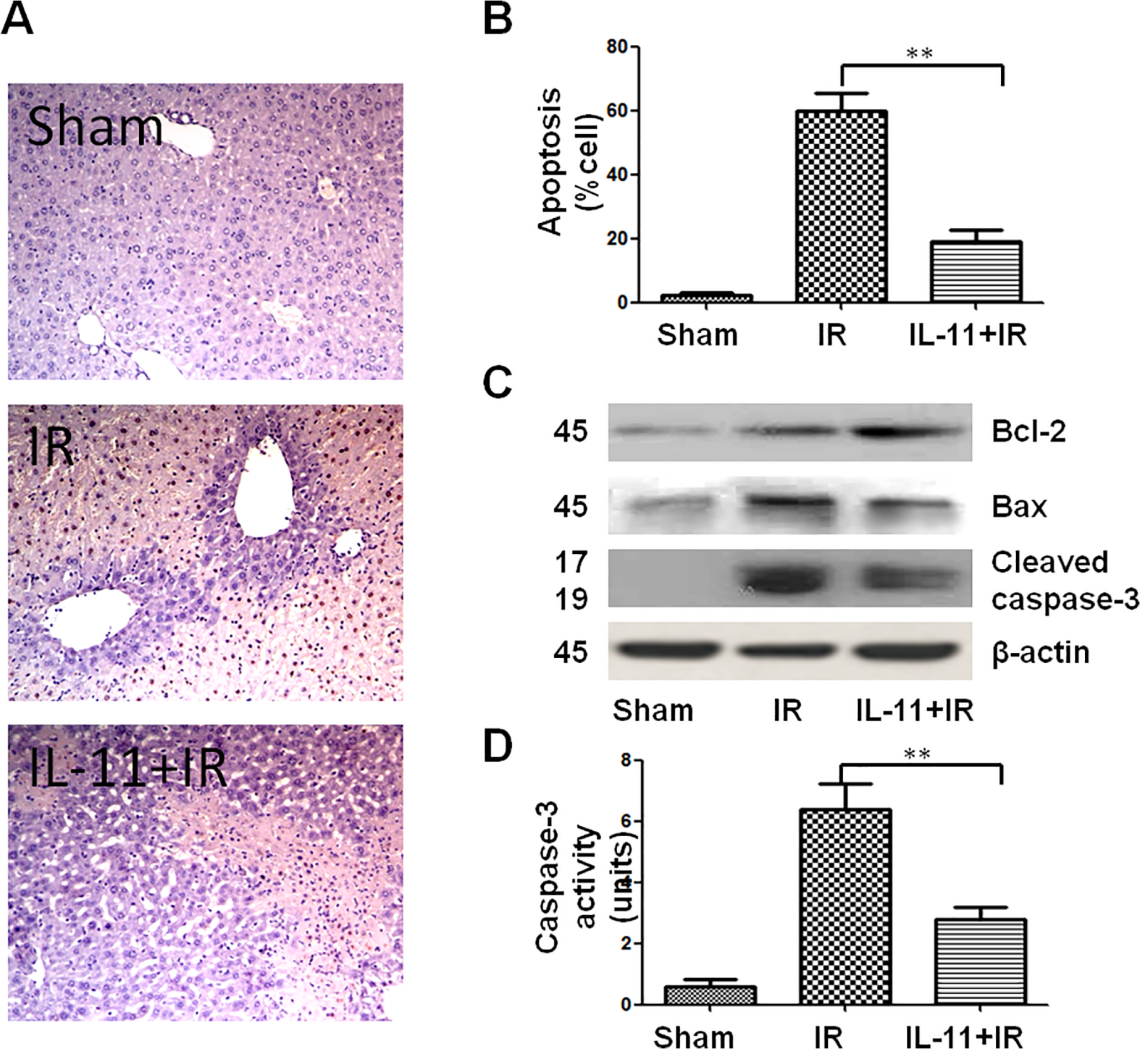
IL-11 treatment decreased hepatocellular apoptosis induced by IR. (A)Liver apoptosis was examined by TUNEL staining: Sham group, IR group and IL-11+IR group. (B) Apoptotic cells were quantified in six high-power fields (400x), and expressed as percentages of apoptotic cells among total cells. (C) Western blot-assisted detection of Bcl-2, Bax, Caspase-3 and β-actin. (D) Caspase-3 activity. Data are expressed as mean±SD (n = 6/group), **P<0.001.

Apoptosis/necrosis is a central mechanism of cell death in liver IRI. In this study, hepatocellular apoptosis was analyzed in ischemic livers by TUNEL staining 6 hours after reperfusion. Our results showed TUNEL-positive cells were significantly lower in liver sections of the IL-11 treatment group compared with those in IR control group (Fig 4A and 4B). TUNEL-positive cells in the total hepatocytes of the three groups were (0.60±0.25)%, (10.20±1.28)%, and (4.00±0.63)%, respectively, indicating that hepatocellular apoptosis was significantly reduced by IL-11 administration. In addition, anti-apoptotic protein Bcl-2 and pro-apoptotic protein Bax were analyzed in ischemic liver, which showed that IL-11 treatment effectively upregulated Bcl-2 and inhibited Bax expression (Fig 4C). Caspase-cascade system, especially the component Caspase-3, plays a critical role during liver IRI. Caspase-3 affects apoptosis by component cleaved Caspase-3. Next, we assessed the expression of cleaved Caspase-3 by immune analysis, suggesting that IL-11 administration effectively inhibited expression of cleaved Caspase-3 during liver IRI (Fig 4C). Apoptotic active caspase-3 directly caused hepatocellular apoptosis and reflected the progress of apoptosis in the ischemic liver. Along with the TUNEL assay and expression of cleaved Caspases-3, Fig 4D shows that the activity of caspase-3 was significantly repressed after IL-11 preconditioning in ischemic liver tissue compared with the IR group (1.38±0.08 and 4.44±0.289, respectively; *P*<0.001).

### IL-11 regulates inflammatory program and MPO activity in IR-stressed liver

**Fig 5:**
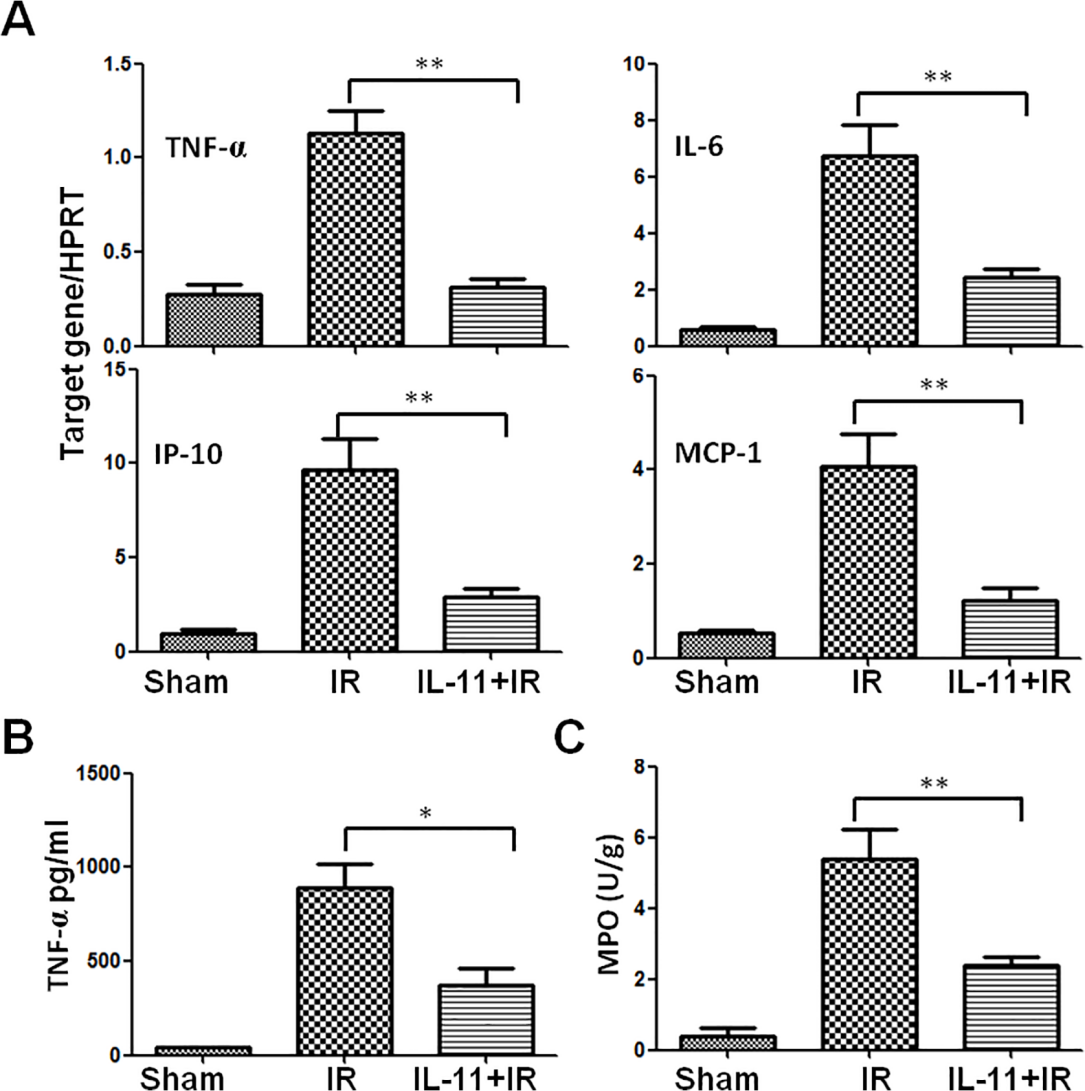
IL-11 treatment inhibited pro-inflammatory responeses induced by IR. (A) Cytokine gene (TNF-a, IL-6, IP-10 and MCP-1) expressions were analyzed in ischemic livers by RT-PCR analysis. Expressions of cytokine gene were normalized with that of HPRT. (B) TNF-α secretion was examined in serum by ELISA. (C) MPO activity in ischemic liver. Data are expressed as mean±SD (n = 6/group), *P<0.05, **P<0.001.

A complex inflammatory program involving cytokines and chemokines is engaged in liver IRI. Inflammatory cytokines (TNF-α and IL-6) and chemokines (IP-10 and MCP-1) displayed proinflammatory and proapoptotic roles in ischemic liver post-reperfusion. To further assess the hepatoprotective effects of IL-11 treatment, mRNA expressions of TNF-α, IL-6, IP-10 and MCP-1 were determined in ischemic liver after 6h of reperfusion by qRT-PCR. Fig 5A shows a significantly lower level of TNF-α (1.13.±0.13 and 0.31±0.04, respectively; *P*<0.001), IL-6 (6.73±1.12 and 2.43±0.33, respectively; *P*<0.001), IP-10 (9.69±1.63 and 2.93±0.39, respectively; *P*<0.001) and MCP-1 (4.08±0.70 and 1.22±0.27, respectively; *P*<0.001) in the IL-11 administration group compared with that in the IR group. These data indicated that IL-11 effectively inhibited the expression of inflammatory cytokines in ischemic liver after reperfusion. In addition, serum TNF-α was further analyzed by ELISA, which showed IL-11 treatment significantly decreased TNF-α secretion after liver IRI (Fig 5B). The protein level was consistent with gene expression. The MPO activity, reflecting neutrophil activity and infiltration, was reduced after reperfusion in IL-11 treated liver compared with controls (5.40±0.81U/g and 2.40±0.25U/g, respectively; *P*<0.001) (Fig 5C).

### Activation of STAT3 is essential for IL-11-mediated protective role

**Fig 6:**
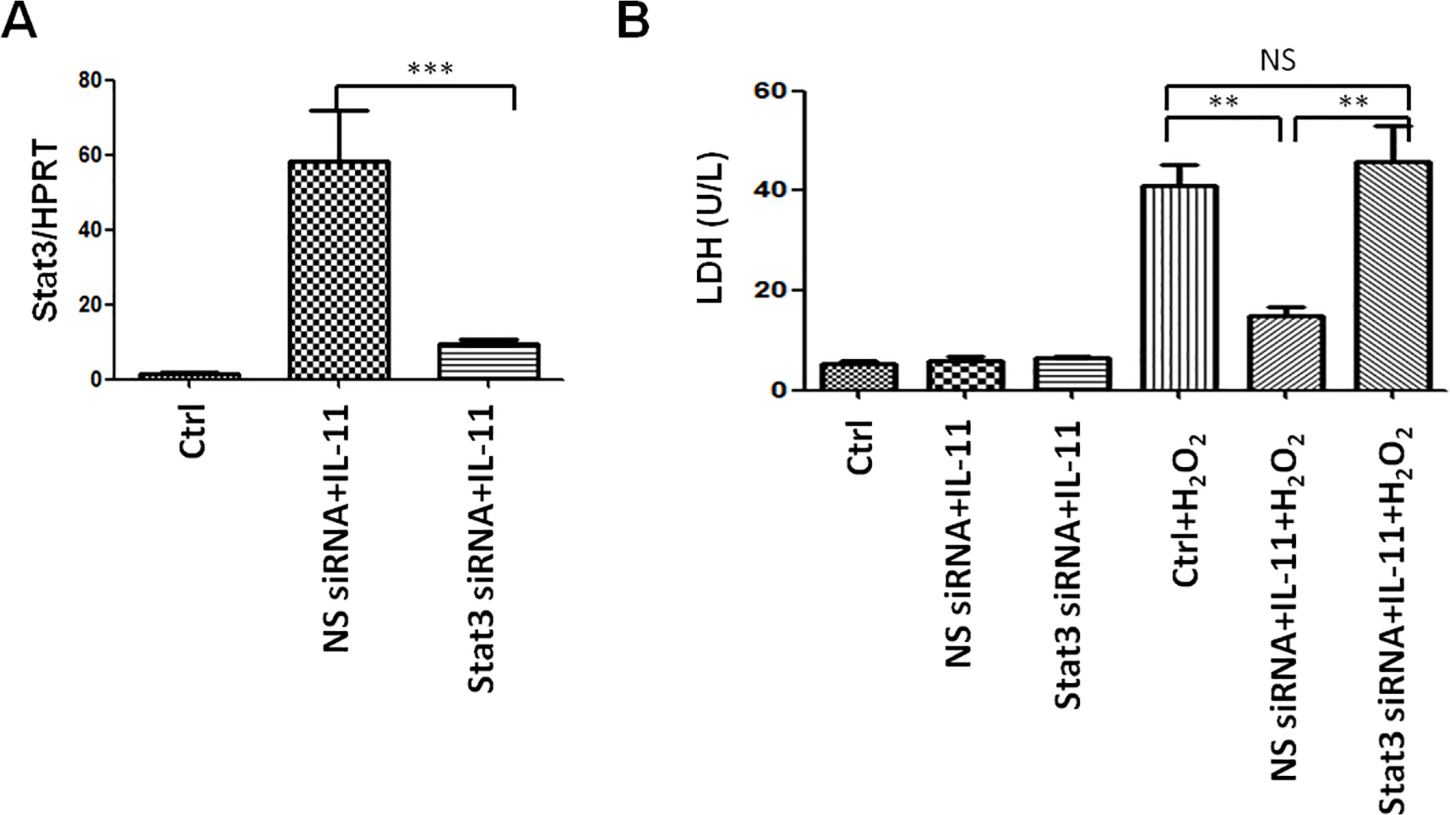
STAT3 knockdown abolished IL-11 protective effects in vitro. (A) STAT3 gene expression was analyzed in transfected hepatocytes by RT-PCR analysis. Expressions of gene were normalized with that of HPRT. (B) The released LDH level of hepatocytes after H_2_O_2_ treatment. Data are expressed as mean±SD (n = 4-6/group), **P<0.001, ***P<0.0001.

To evaluate the effect of STAT3 activation in an IL-11-mediated protective role, primary hepatocytes were transiently transfected with STAT3 siRNA or NS siRNA. Next, STAT3 gene expression was assessed after IL-11 treatment by qRT-PCR, which showed STAT3 gene expression was significantly repressed compared with the NS siRNA control (Fig 6A). These data indicated that STAT3 expression was successfully knocked down in hepatocytes. Then, these transfected cells were treated with H_2_O_2_ to induce cell death. The released LDH level was checked in the supernatant after H_2_O_2_ treatment for 24h. Fig 6B shows that IL-11 treatment remarkably inhibited LDH release of hepatocytes after H_2_O_2_ treatment (14.00±4.32 and 14.80±1.99, respectively; *P*<0.001). However, STAT3 knockdown almost restored the decreased LDH release after IL-11 treatment (14.80±1.99 and 45.80±7.14, respectively; *P*<0.001). These data indicated STAT3 activation is necessary for IL-11-mediated protective role.

### Knockdown of STAT3 restores liver IRI in IL-11-treated mice

**Fig 7:**
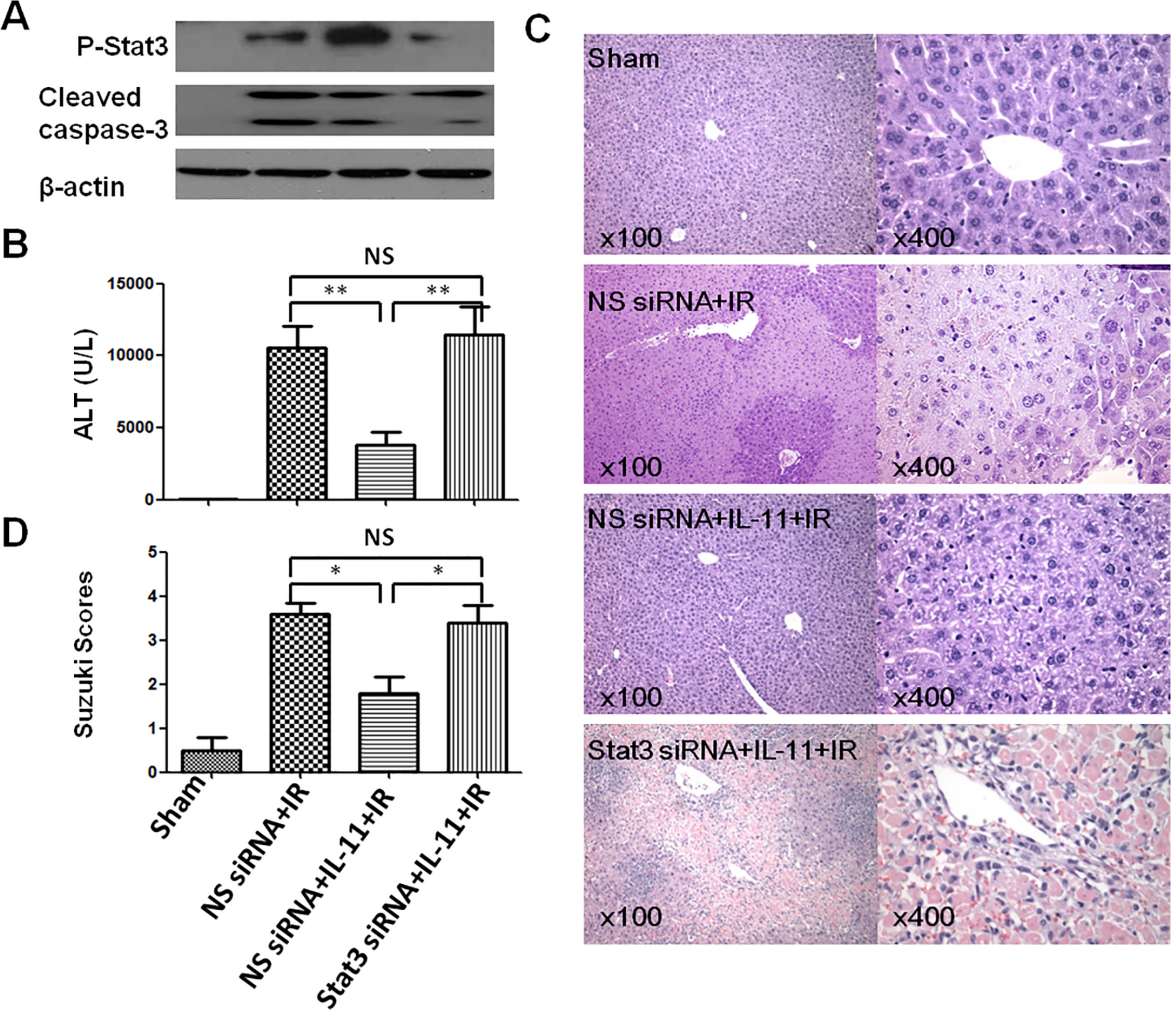
STAT3 knockdown abolished IL-11 protective effects in vivo. Mice were injected with STAT3 siRNA or NS siRNA 4h prior to ischemia, and administered with IL-11 1h prior to ischemia, followed by 6h reperfusion. (A) Western blot-assisted detection of P- STAT3, Cleaved Caspase-3 and β-actin. (B) sALT. (C) Histopathalogic analysis of livers harvested 6h after reperfusion. Sham group: Normal hepatic architecture; NS siRNA+IR group: severe hepatic lobule distortion, sinusoidal congestion, apparent edema, vacuolization and massive necrosis; NS siRNA+IL-11+IR group: mild vacuolization, punctate necrosis and edeman; STAT3 siRNA+IL-11+IR group: severe hepatic lobule distortion, sinusoidal congestion, apparent edema, vacuolization and massive necrosis. (D) The severity of liver IRI by Suzuki’s histological grading. Data are expressed as mean±SD (n = 6/group), *P<0.05.

To further assess the effect of STAT3 activation in IL-11-mediated hepoto-protective role during liver IRI, mice were injected intravenously with STAT3 siRNA or NS siRNA prior to ischemia. As shown in Fig 7A, IL-11 treatment significantly increased expression of P-STAT3, which was reversed in STAT3 siRNA group. Fig 7B shows STAT3 siRNA treatment effectively neutralized the reduced sALT levels after IL-11treatment (*P*<0.001) (3795±879U/L versus 11460±1941U/L, respectively). These data correlated with Suzuki’s histological grading of liver IRI (Fig 7C and 7D). Indeed, IL-11 resulted in minimal liver sinusoidal congestion, vacuolization without edema, or necrosis [Fig 7C (c); score = 1.80±0.37]. In contrast, livers in untreated or STAT3 siRNA-treated mice displayed moderate to severe edema and extensive hepatocellular necrosis [Fig 7C (b and d), Fig 7D (panels b and d); score = 3.60±0.25 and 3.40±0.40, respectively]. In addition, cleaved Caspase-3 was further analyzed in ischemic liver, which showed IL-11 treatment partially inhibited IR-triggered cleaved Caspase-3 upregulataion (Fig 7A). These data indicated knockdown of STAT3 recreates liver IRI in IL-11-treated mice.

## Discussion

IL-11 has displayed protective roles during ischemia-reperfusion injury (IRI) in the heart, kidney and intestine, but a similar role has not been elucidated in liver IRI [12, 13, 22, 23]. In this study, we have for the first time demonstrated that IL-11 pretreatment is a promising method for preventing or minimizing liver IRI. Indeed, IL-11 treatment improved liver function, attenuated histology damage, impaired proinflammatory cytokine/chemokine programs, and reduced hepatocellular death/apoptosis in IR-stressed livers. However, the IL-11 mediated protective role was partially impaired by STAT3 siRNA in vivo and in vitro. These findings demonstrate that IL-11 treatment attenuates liver IRI through activating the STAT3 signaling pathway.

IL-11 is a hematopoietic IL-6 family cytokine first identified from marrow-derived stromal cells. It is a key regulator of hematopoiesis and promotes megakaryocyte maturation [24]. IL-11 as well as its receptors is expressed in many tissues and cell types, including macrophages, hepatocytes, etc. in liver tissues [25]. Du’s group for the first time reported the protective effects of IL-11 in models of cytoablative chemoradiotherapy in 1994. Then, some researchers have demonstrated that IL-11 play protective roles in various pathophysiologic states [25]. Different from other IL-6 family members, IL-11 administration reduces inflammatory responses in chronic inflammatory diseases, lipopolysaccharide-induced sepsis, macrophages inflammation, nephrotoxic nephritis and T-cell mediated liver injury [9–11]. The present study also demonstrated that IL-11 treatment effectively inhibited inflammatory responses of ischemic liver, as evidenced by reducing pro-inflammatory cytokines (TNF-α and IL-6) and chemokines (IP-10 and MCP-1), and repressing MPO activtity. In addition to its anti-inflammatory properties, IL-11 treatment has been shown to attenuate necrotic and apoptotic cell death in many organs including the heart, intestines and endothelial cells [25]. In fact, apoptosis/necrosis is a key mechanism of cell death in liver IRI, which directly indicates the extent of liver damage. In our study, hepatocellular death/necrosis as well as apoptosis was also observed in ischemic liver. Our data showed that IL-11 attenuated hepatocellular death/necrosis (ALT, Suzuki’s score) as well as apoptosis (TUNEL staining, cleaved-Caspase 3 fragmentation) in ischemic liver after IRI. In addition, we also analyzed effects of IL-11 on hepatocellular death/necrosis by LDH release level in vitro, which was consistent with in vivo results. Thus, we conclude that IL-11 administration provides liver protection against IRI by reducing inflammation and necrosis/apoptosis.

The molecular mechanisms of IL-11 mediated signaling pathway for an anti-inflammatory and anti-necrosis/apoptosis might involve the activation of multiple intercellular pathways. After IL-11 binds to the IL-11 receptor, the ligand-receptor complex interacts with a common receptor subunit, glycoprotein 130 (gp130), leading to gp130-associated kinase-mediated tyrosine phosphorylation [26]. In vascular endothelial and intestinal epithelial cells, IL-11 protects against oxidant induced necrosis and apoptosis via mechanisms involving ERK, MAPK, AKT and/or induction of HSP25 [22, 27, 28]. In renal IRI, IL-11 performs renal protection by direct induction of sphingosine kinase-1 (SK-1) via nuclear translacation of HIF-1α. In cardiac myocytes, IL-11 treatment attenuates injury and fibrosis via Janus Kinase-Signal Transducer and Activation of Transducer 3 (JAK-STAT3) pathway activation [12, 23, 29]. Kawakami T et al have demonstrated rhIL-11 confers significant protection against CCl4-induced hepatic injury by virtue of its liver-specific HO-1 induction [30]. In addition, a previous studydemonstrated STAT3 activation after Ad-HO-1 treatment improved the hepatocellular function in a mouse model of segmental liver warm IRI [31]. In the present study, IL-11 treatment rapidly activated STAT3 in hepatocytes in vivo and in vitro, and reduced liver injury after reperfusion. However, IL-11 administration shows weak hepatocellularprotective effects in STAT3siRNA transfected hepatocytes or STAT3siRNA transfected mice. Therefore, hepatocyte is an important target in the action of IL-11, and IL-11-mediated protection of liver IRI is partially dependent on STAT3 activation of hepatocytes. Whether HO-1 is involved in this protective procedure will be further investigated in the future.

Evidence exists that the STAT3 signaling pathway transduces stress-activating extracellular chemical signals into cellular responses for a number of pathophysiological processes, such as immunity, inflammation and apoptosis, and is involved in liver IRI. The function of activated STAT3 is controversial; some studies have associated it with survival [31–33], while others have related it to cell death [34]. Previous studies have confirmed that STAT3 alterations affect Bcl-2 and Bax protein expression and induce inflammation and apoptosis in many types of tumor cells [35–37]. In mycosis fungoides tumor cells, some apoptosis-related genes, such as Bcl-2 and Bax, have been identified as STAT3 target genes [38]. In our study, STAT3 activation reduced hepatocellular necrosis/apoptosis and liver injury induced by IR after IL-11 treatment, but STAT3 knockdown restored the hepatocellular necrosis/apoptosis and liver injury in IL-11-treated mice. These data indicate that IL-11 treatment reduces hepatocellular necrosis/apoptosis by STAT3 activation. Lee et al demonstrated that co-activated NF-κB and STAT3 modulate Bax/Bcl-xL expression and promote cell survival in head and neck squamous cell carcinoma [38]. In primary cortical neurons and murine models of stroke, the activation of STAT3 pathway by secretoneurin has been found to exert neuroprotective effects and induce neuronal plasticity after hypoxia and ischemic insult [39]. Using a mouse model of myocardial infarction, Obana M et al demonstrated that IL-11 exerted protective effects against myocardial ischemic injury through IL-11R-mediated STAT3 activation, antiapoptotic signaling and proangiogenic activity [23]. In addition, STAT3 has been demonstrated to have an anti-inflammatory function in many pathophysiological processes [23]. Inflammatory response plays a pathogenic role in liver I/R injury, especially innate immune responses involved in cytokines and chemokines, including TNF-α, IL-6, IP-10, MCP-1 and so on [40–43]. Ke B et al demonstrated that STAT3 activation repressed TLR4-drived inflammation by activating PI3K/Akt signaling during liver IRI [23]. Consistent with the above data, our results also showed that IL-11-induced STAT3 signaling inhibited pro-inflammatory cytokines and chemokines.

In conclusion, our findings demonstrate for the first time IL-11-mediated STAT3 attenuates IR-triggered liver injury. IL-11-mediated STAT3 signaling not only reduces hepatocellular apoptosis, but also inhibits inflammatory responses. Thus, our study provides a rationale for novel therapeutic approaches to the management of liver injury triggered by IR.
